# Supplementary figures and images for: Dynamic and Ballistic Performance of Graphene Oxide Functionalized Curaua Fiber-Reinforced Epoxy Nanocomposites
Source: Polymers (Basel). 2022 May 1;14(9):1859. doi: 10.3390/polym14091859 (PMC9105903; doi:10.3390/polym14091859)

Supplementary information

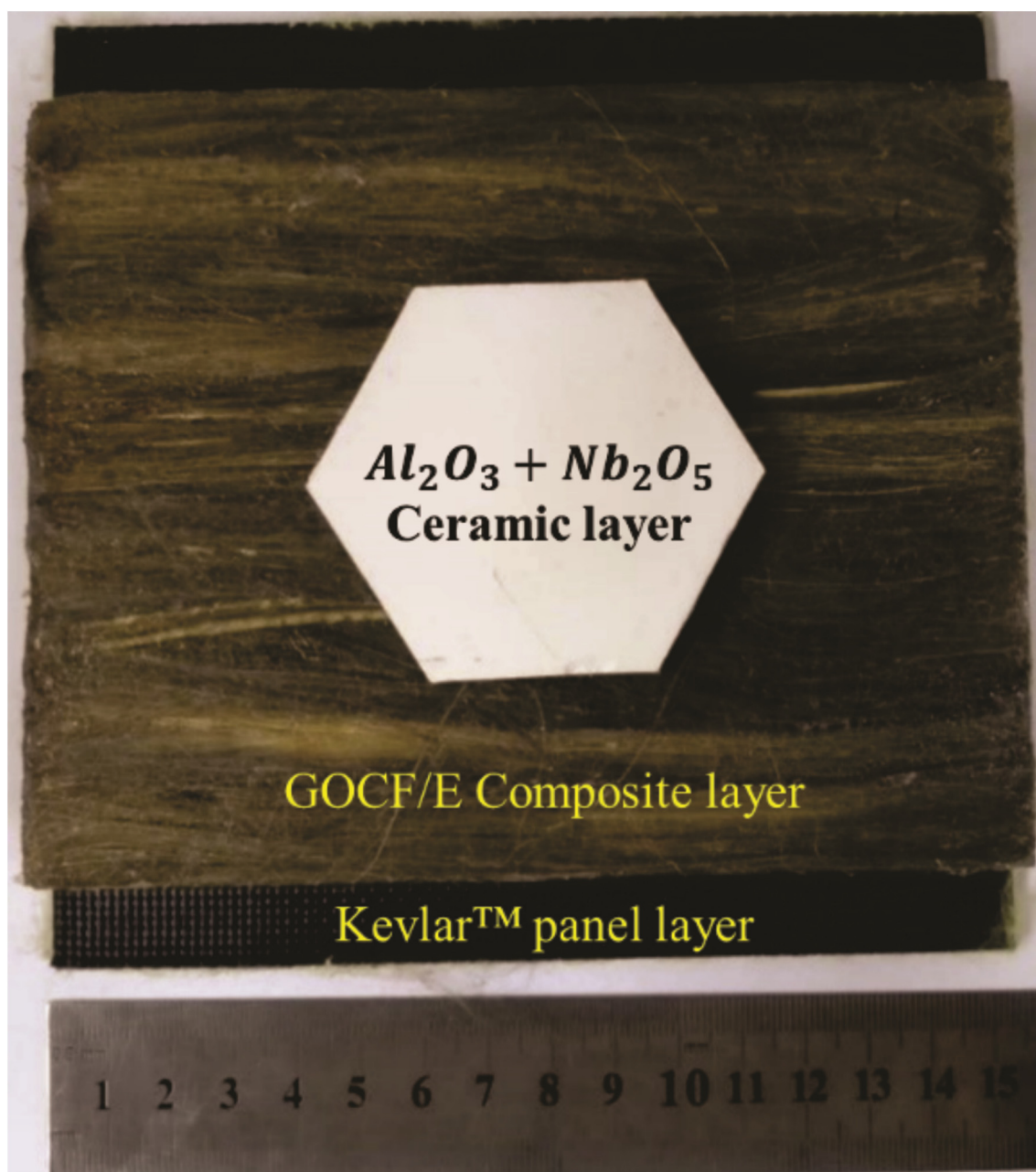

Figure S1. MAS assembly of GOCF/E composite.

Supplement: Supplementary file 1 [file polymers-14-01859-s001.zip › polymers-1638572-supplementary.pdf]
